# Atg5-dependent autophagy contributes to the development of acute myeloid leukemia in an MLL-AF9-driven mouse model

**DOI:** 10.1038/cddis.2016.264

**Published:** 2016-09-08

**Authors:** Qiang Liu, Longgui Chen, Jennifer M Atkinson, David F Claxton, Hong-Gang Wang

**Affiliations:** 1 Department of Pharmacology, Penn State College of Medicine, Hershey, PA, USA; 2 Department of Pediatrics, Penn State College of Medicine, Hershey, PA, USA; 3 Penn State Hershey Cancer Institute, Penn State College of Medicine, Hershey, PA, USA

## Abstract

Acute myeloid leukemia (AML) is a hierarchical hematopoietic malignancy originating from leukemic stem cells (LSCs). Autophagy is a lysosomal degradation pathway that is hypothesized to be important for the maintenance of AML as well as contribute to chemotherapy response. Here we employ a mouse model of AML expressing the fusion oncogene MLL-AF9 and explore the effects of Atg5 deletion, a key autophagy protein, on the malignant transformation and progression of AML. Consistent with a transient decrease in colony-forming potential *in vitro*, the *in vivo* deletion of Atg5 in MLL-AF9-transduced bone marrow cells during primary transplantation prolonged the survival of recipient mice, suggesting that autophagy has a role in MLL-AF9-driven leukemia initiation. In contrast, deletion of Atg5 in malignant AML cells during secondary transplantation did not influence the survival or chemotherapeutic response of leukemic mice. Interestingly, autophagy was found to be involved in the survival of differentiated myeloid cells originating from MLL-AF9-driven LSCs. Taken together, our data suggest that Atg5-dependent autophagy may contribute to the development but not chemotherapy sensitivity of murine AML induced by MLL-AF9.

Acute myeloid leukemia (AML) is a clonal hematopoietic malignancy characterized by the uncontrolled proliferation of immature myeloid cells within the bone marrow (BM), eventually suppressing normal hematopoiesis.^1^ Recurrent chromosomal translocations frequently occur in AML, one of which involves the fusions of the KMT2A gene on chromosome 11 to a number of potential partners that are diagnosed as prognostically intermediate to poor.^1^ Among these fusions, the MLL-AF9 fusion oncogene, resulting from the t(9;11)(p22;q23) translocation, is well studied owing to its robust phenotype in various mouse models of AML.^2, 3, 4^ It has been previously reported that BM transplantation of hematopoietic progenitors expressing exogenous MLL-AF9 leads to rapid *in vivo* transformation and progression of AML in a syngeneic, immunocompetent mouse model and recapitulates the poor chemotherapy response of t(9;11)(p22;q23) fusion human AML.^2, 5^

Autophagy is an evolutionarily conserved catabolic pathway by which cellular components are engulfed by double-membraned vesicles, called autophagosomes, and delivered to the lysosome for degradation and recycling. Autophagy is best characterized to be induced under stressful conditions, such as organelle damage or nutrient deprivation, and is followed by the elongation of the autophagosome membrane around its cargo. In Atg5-dependent autophagy, the conversion of LC3-I to LC3-II by lipidation is crucial for autophagosome membrane expansion, which is mediated by a series of ubiquitin-like conjugation systems.^6^ Within this pathway, the Atg5-Atg12-Atg16 complex acts as an E3-ubiquitin-ligase-like enzyme that specifically mediates the conjugation of LC3-I to phosphatidylethanolamine to form LC3-II, which inserts to the autophagosomal membrane. Autophagosome maturation is followed by fusion to lysosomes, at which time the inner compartment is degraded. The genetic ablation of Atg5 leads to a complete and highly selective inhibition of LC3-dependent autophagosome formation.^6, 7^

Autophagy is known to be implicated in cancer as both a tumor promoter and a tumor suppressor.^8^ The genetic ablation of autophagy in mouse hematopoietic stem cells (HSCs) has been shown to result in severe impairments to HSC maintenance.^9, 10, 11, 12, 13^ Autophagy dysregulation has also been implicated in AML,^12, 13, 14^ suggesting that targeting autophagy could be promising for AML treatment. As an expanding arsenal of pharmacological autophagy modulators are being developed,^15, 16^ it has become increasingly important to specifically determine whether autophagy has an important role in AML using a genetic mouse model. Therefore, we sought to dissect the role of autophagy through the *in vivo* homozygous deletion of Atg5 in MLL-AF9-driven murine AML. We discover in this study that Atg5 deletion during primary transplantation prolongs the survival of animals, whereas Atg5 deletion after secondary transplantation has no effect on animal survival, suggesting a role for autophagy in the initiation, but not maintenance, of AML in our model. We additionally assessed the effect of autophagy in chemotherapeutic response and found that Atg5 deletion in our MLL-AF9 model had no effect on the *in vivo* response to cytarabine and doxorubicin combination therapy, suggesting that autophagy does not significantly contribute to chemotherapy response in this model.

## Results

### A dual-promoter/reporter MLL-AF9 vector enables leukemogenesis and non-invasive bioluminescent imaging to assay Atg5-dependent autophagy

**Figure 1:**
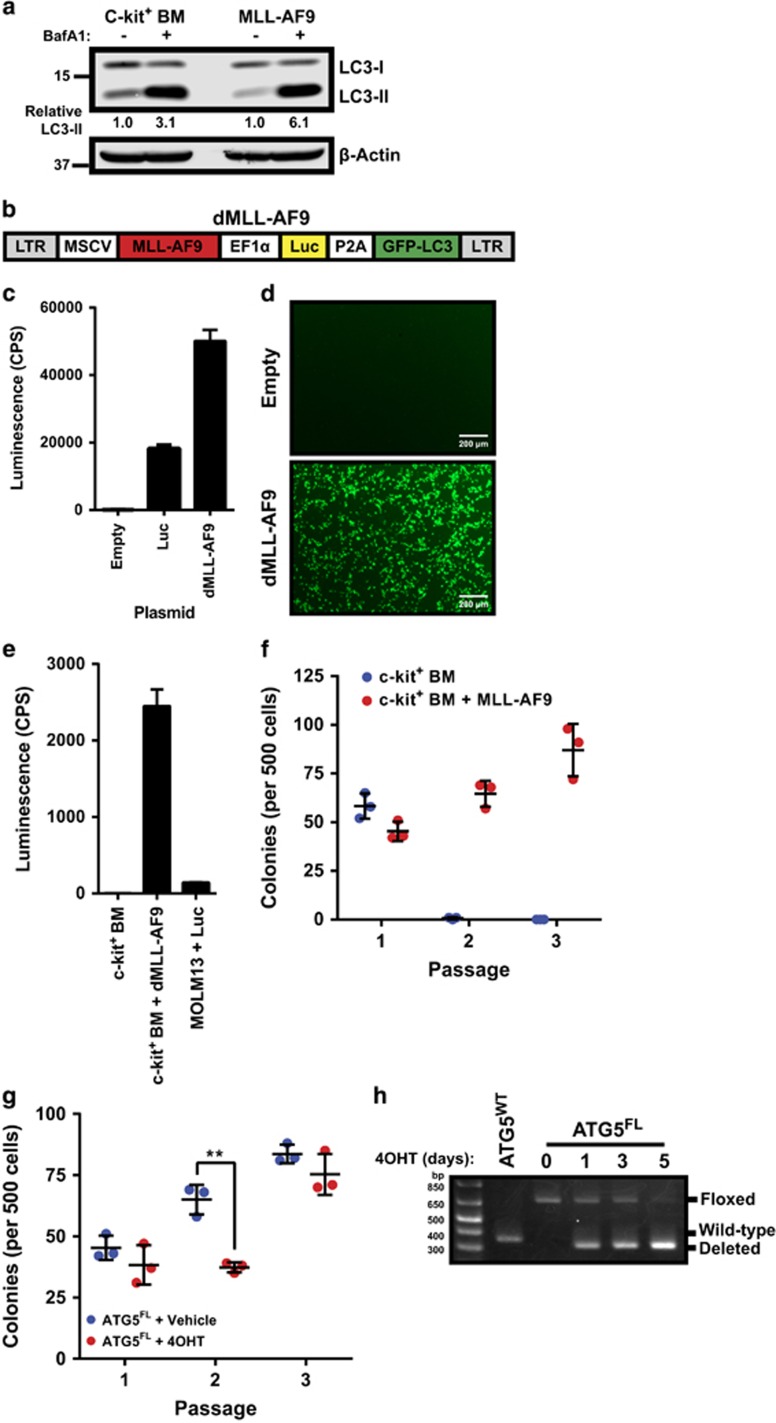
Verification of the dMLL-AF9 vector expressing luciferase and GFP-LC3. (**a**) C-kit^+^ BM cells or malignant leukemia cells driven by exogenous pMIG-MLL-AF9 were cultured in cytokine-supplemented medium and treated with vehicle or 100 nM BafA1 for 6 h and subjected to immunoblotting. Quantified LC3-II levels were normalized against *β*-actin and its respective vehicle-treated control. Western blotting is representative of two independent experiments. (**b**) Schematic representing the retroviral construct containing MLL-AF9, luciferase (Luc), and GFP-LC3 with their respective promoters. (**c**) The luminescence of PHOENIX/Eco cells 24 h after calcium transfection of the pMSCV-empty (Empty), pMSCV-Luc-IRES-YFP (Luc), or dMLL-AF9. Error bars represent S.D. of three replicates. (**d**) Green fluorescence of PHOENIX/Eco cells 24 h after calcium transfection with the indicated vectors was detected with the Olympus CKX41 microscope using the Olympus DP20 camera and the Olympus CellSens software (original magnification × 40). (**e**) Luminescence of non-transduced or dMLL-AF9-transduced c-kit^+^ BM cells (c-kit^+^ BM+dMLL-AF9) compared with MOLM13 cells stably expressing pMSCV-Luc-IRES-YFP (MOLM13+Luc). Error bars represent S.D. of three replicates. (**f**) Non-transduced or dMLL-AF9 transduced c-kit^+^ BM cells were seeded to methylcellulose for three passages at 5 days each and counted for the number of colonies. Error bars represent S.D. of three replicates. (**g**) One day after c-kit^+^ BM were transduced with dMLL-AF9, cells were seeded to methylcellulose medium containing 100 nM 4OHT for three rounds of serial replating. Black bars represent the mean±S.D. Statistics were calculated by ANOVA with multiple comparisons. **P<0.01. (**h**) Atg5^FL^ c-kit^+^ BM were treated with 100 nM 4OHT for the indicated days, and control Atg5^WT^ c-kit^+^ BM cells were treated with 100 nM 4OHT for 5 days. Genomic DNA was extracted and analyzed by PCR

The extent of LC3-II accumulation under autophagic flux inhibition is a marker for the level of Atg5-dependent autophagy. Bafilomycin A1 (BafA1), an inhibitor of the vacuolar H^+^ ATPase, blocks lysosomal degradation and eventually prevents autophagosome fusion with lysosomes.^7^ Autophagy has been thought to be dysregulated in AML, suggesting a potentially important role for autophagy in AML pathogenesis.^8, 13, 14^ The level of autophagic flux under basal conditions was therefore measured in malignant murine AML cells expressing exogenous MLL-AF9, compared with their healthy BM counterpart *in vitro*. Compared with vehicle-treated conditions, BafA1 treatment resulted in greater LC3-II accumulation in MLL-AF9 cells compared with c-kit^+^ BM (Figure 1a). The quantification of normalized LC3-II levels revealed that MLL-AF9^+^ leukemia cells (6.1) have higher levels of autophagic flux compared with c-kit^+^ BM (3.1).

In order to facilitate the non-invasive monitoring of disease progression *in vivo* and the fluorescent imaging of autophagy, we designed a dual-promoter/reporter MLL-AF9 retroviral vector (hereafter referred to as dMLL-AF9) for MLL-AF9-dependent leukemogenesis and non-invasive monitoring of disease progression in mice (Figure 1b). The MLL-AF9 oncogene is driven by the murine stem cell virus (MSCV) promoter, and the firefly luciferase was cloned under the elongation factor-1 alpha (EF1*α*) promoter, which has been demonstrated to express strongly in hematopoietic cells.^17^ GFP in traditional leukemogenic vectors was substituted with GFP-LC3 in order to monitor autophagy. Both cistrons were joined with a self-cleaving P2A motif, which would result in the translational cleavage between the two reporters and enforces strict one-to-one expression.^18^ We confirmed that all cistrons were functional in the dMLL-AF9 vector. Both luciferase activity and green fluorescence were readily detected in HEK293T cells transiently transfected with dMLL-AF9 (Figures 1c and d). Luciferase activity was also detected strongly in c-kit^+^ BM transduced with dMLL-AF9, compared with non-transduced c-kit^+^ BM or the human AML cell line MOLM13 stably expressing luciferase (Figure 1e). In order to determine whether the MLL-AF9 oncogene was functional, we confirmed that murine c-kit^+^ BM cells transduced with dMLL-AF9 were immortalized for three passages of *in vitro* methylcellulose culture, whereas healthy c-kit^+^ BM demonstrated limited colony-forming potential beyond the first passage (Figure 1f).

### Atg5 deletion during primary transplantation extends the survival of mice burdened with MLL-AF9-driven AML

In order to specifically assess the *in vivo* role of autophagy in the malignant transformation and progression of AML, we took advantage of transgenic mice expressing a tamoxifen-inducible Cre recombinase (CreERT2)^19^ in combination with floxed Atg5 alleles (Atg5^FL/FL^). Therefore, the transplantation of cells from donor mice with the genotypes of Atg5^WT/WT^;CreERT2^+/−^ (Atg5^WT^) and Atg5^FL/FL^;CreERT2^+/−^ (Atg5^FL^) to congenic C57BL/6J recipients followed by tamoxifen treatment induces the specific *in vivo* deletion of Atg5 in Atg5^FL^ donor cells.

To determine whether Atg5 deletion affects MLL-AF9-induced transformation of hematopoietic progenitors, we transduced c-kit^+^ Atg5^FL^ BM cells with dMLL-AF9 retrovirus and subjected them to a serial colony-forming unit assay in methylcellulose containing 4-hydroxytamoxifen (4OHT) to delete Atg5 *in vitro*. When compared with vehicle control, Atg5^FL^ cells treated with 4OHT demonstrated a transient but significant decrease in colony-formation potential during the second passage (Figure 1g), suggesting that autophagy may contribute to MLL-AF9-driven leukemogenesis. We confirmed that a 5-day continuous treatment with 4OHT is required to complete Atg5 deletion (Figure 1h).

**Figure 2:**
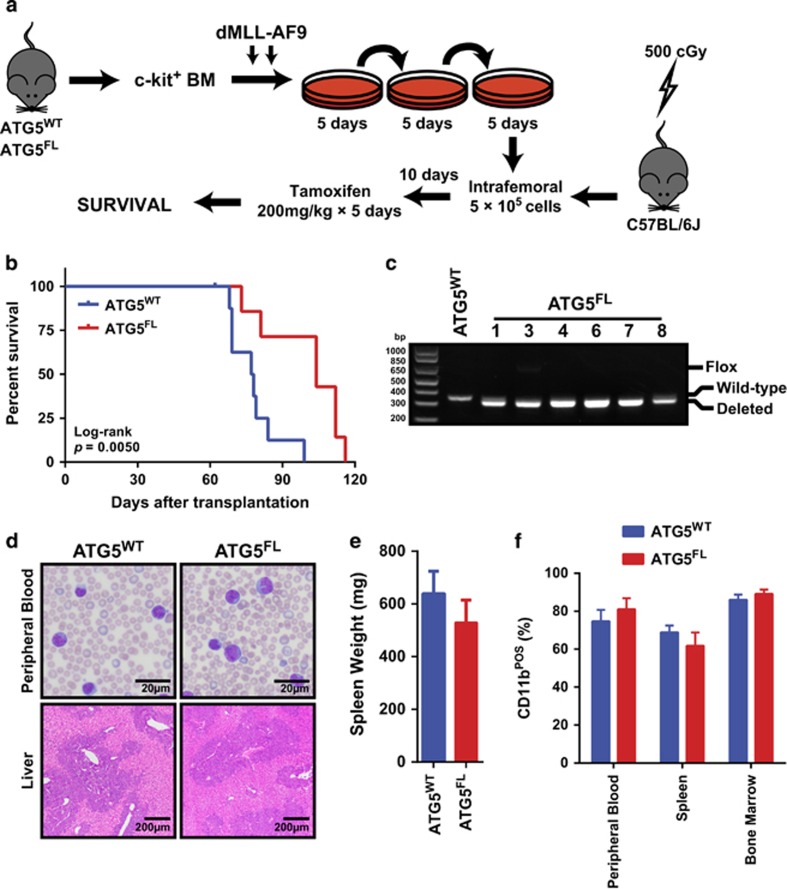
Development of AML in mice transplanted with ATG5^WT^ and Atg5^FL^ MLL-AF9-BM cells transduced with dMLL-AF9 and treated with tamoxifen. (**a**) A schematic representing the strategy by which the role of Atg5-dependent autophagy was assessed during primary transplantation in MLL-AF9-driven AML. (**b**) The Kaplan–Meier survival curve of mice transplanted with Atg5^WT^ (*n*=8) and Atg5^FL^ (*n*=7) MLL-AF9 cells. The *P*-value for the log-rank test between the two groups is shown. (**c**) Genomic DNA extracted from the splenocytes of a representative Atg5^WT^ and six Atg5^FL^ moribund mice from panel (**b**) were analyzed by PCR for the status of Atg5 alleles. (**d**) Representative figures of peripheral blood smears stained by May Grünwald–Giemsa (top) and liver section stained by hematoxylin and eosin (bottom) of moribund mice from panel (**b**). (**e**) The spleen weight of moribund mice from panel (**b**) for Atg5^WT^ (*n*=7) and Atg5^FL^ (*n*=6) mice. Error bars represent S.E.M. (**f**) The Annexin V^−^7-AAD^−^CD11b^+^ myeloid cells are shown as percentage of all Annexin V^−^7-AAD^−^ cells according to flow cytometry in the indicated hematopoietic tissues of moribund mice from panel (**b**). Error bars represent S.E.M. of six mice

Next we assessed the role of autophagy in MLL-AF9-driven AML development and progression *in vivo*. Atg5^WT^ and Atg5^FL^ c-kit^+^ BM cells were transduced with dMLL-AF9 and expanded for three rounds of methylcellulose culture (Figure 2a). Cells were then intrafemorally transplanted to sublethally irradiated C57BL/6J recipients and treated with tamoxifen after 10 days in order to assess the role of Atg5 during primary transplantation. Mice transplanted with Atg5^WT^ or Atg5^FL^ BM cells expressing MLL-AF9 became moribund with a median survival of 77.5 days and 104 days, respectively, demonstrating a prolonged survival for the Atg5^FL^ group of mice (Figure 2b). PCR confirmed that Atg5 was efficiently deleted in the splenocytes of mice transplanted with Atg5^FL^ cells (Figure 2c). We observed evidence of anemia and myeloid blasts in peripheral blood, as well as significant perivascular infiltration of blasts to the liver for both groups of mice (Figure 2d). Splenomegaly was consistently observed and no differences were observed between the two groups at morbidity (Figure 2e). Flow cytometric analysis of the peripheral blood, spleen, and BM revealed that all three hematopoietic tissues show high percentages of CD11b^+^ myeloid cells (Figure 2f). No differences were observed in blast morphology, liver infiltration, or myeloid cell proportions between mice transplanted with Atg5^WT^ and Atg5^FL^ cells at morbidity. This data suggest that autophagy facilitates the initiation of MLL-AF9-driven AML in our model.

**Figure 3:**
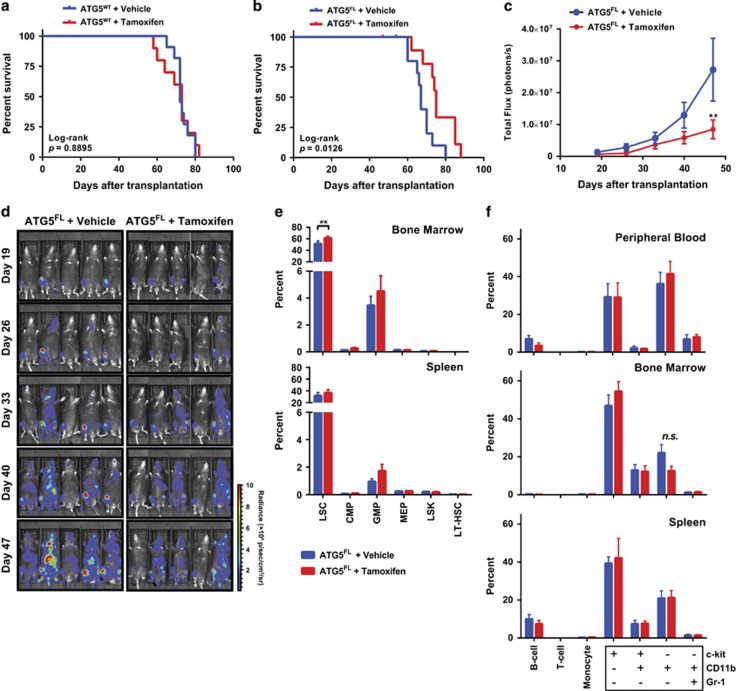
Vehicle and tamoxifen treatment in mice transplanted with ATG5^WT^ and Atg5^FL^ BM cells transduced with dMLL-AF9. (**a** and **b**) Kaplan–Meier survival curve of mice transplanted with Atg5^WT^ (**a**) or Atg5^FL^ (**b**) cells treated with vehicle or tamoxifen (*n*=11 for each group). The *P*-values for the log-rank test between vehicle and tamoxifen treatment are shown. (**c**) Quantification of *in vivo* bioluminescent imaging of Atg5^FL^ mice from panel (**b**) is represented by total flux. Error bars represent S.E.M. of nine mice. Statistics were calculated by analysis of variance (ANOVA) with multiple comparisons. **P<0.01. (**d**) Representative animals for *in vivo* bioluminescence of mice from panel (**b**). Images were normalized to the scale bar at bottom right. (**e**) Flow cytometric analysis of Annexin V^−^7-AAD^−^ cells in the indicated hematopoietic tissues of moribund mice from panel (**b**), showing the following cell populations: c-kit^+^Sca-1^−^CD16/32^+^CD34^−^ LSCs, c-kit^+^Sca-1^−^CD16/32^−^CD34^+^ CMPs, c-kit^+^Sca-1^−^CD16/32^+^CD34^+^ GMPs,c-kit^+^Sca-1^−^CD16/32^−^CD34^−^ MEPs, Lin^−^c-kit^+^Sca-1^+^ LSKs, and Lin^−^c-kit^+^Sca-1^+^CD48^−^CD150^+^ LT-HSCs. Error bars represent S.E.M. of eight mice. Statistics were calculated by ANOVA with multiple comparisons. **P<0.01. (**f**) Flow cytometric analysis of Annexin V^−^7-AAD^−^ cells in the indicated hematopoietic tissues of moribund mice from panel (**b**) shows the populations of CD19^+^ B-cells, CD3^+^ T-cells, F4/80^+^ monocytes, and cells of the indicated phenotype. Statistics were calculated by ANOVA with multiple comparisons. NS, not statistically significant

In order to overcome experimental limitations regarding stem cell heterogeneity^20^ and oncogene dosage,^21^ as well as to control for toxicities associated with Cre recombinase^22^ and tamoxifen,^23^ we performed a series of experiments where mice were transplanted with Atg5^WT^ or Atg5^FL^ donor cells transduced with dMLL-AF9, each followed by vehicle or tamoxifen treatment. Mice transplanted with a single pool of Atg5^WT^ cells and treated with vehicle or tamoxifen succumbed to AML without a significant difference in median survival, suggesting that active Cre recombinase or tamoxifen does not affect the survival of leukemic mice in the absence of Atg5 deletion (Figure 3a). However, mice transplanted with a single pool of Atg5^FL^ cells expressing dMLL-AF9 and treated with either vehicle or tamoxifen demonstrated a modest but significant survival advantage for tamoxifen-treated mice (Figure 3b), consistent with our previous findings (Figure 2b). Weekly *in vivo* bioluminescent imaging of these Atg5^FL^ mice revealed that total leukemia burden was decreased over time in Atg5^FL^ mice treated with tamoxifen relative to vehicle-treated mice (Figures 3c and d). Therefore, we concluded that the prolonged survival of mice burdened with AML following primary transplantation is an effect specifically associated with Atg5 deletion.

We examined various hematopoietic progenitor populations in moribund Atg5^FL^ mice to determine whether LSCs were affected by Atg5 deletion. LSCs were previously characterized to be exclusively within the c-kit^+^Sca-1^−^ population in this model of MLL-AF9-driven AML, where CD16/32 is highly enriched but CD34 expression is dispensable.^2, 24^ Data from our laboratory has shown that the c-kit^+^Sca-1^−^ population of MLL-AF9-induced AML cells is almost exclusively CD16/32^+^ and CD34^−^ (unpublished observations) and we therefore refer to these cells as phenotypic LSCs. We observed the number of LSCs to be higher in the BM of tamoxifen-treated animals but not in the spleen (Figure 3e). Other progenitor cell populations^25^ were not found to be different between the vehicle- and tamoxifen-treated mice. Atg5 deletion also had no effect on lymphocyte, F4/80^+^ monocytes, or c-kit^−^ myeloid cells in the peripheral blood, BM, or spleen at morbidity (Figure 3f). Thus, although *in vivo* Atg5 deletion during primary transplantation resulted in prolonged survival, leukemic mice lacking Atg5 are similar in disease presentation at morbidity compared with mice with intact Atg5.

### Atg5 ablation increases apoptosis of differentiated malignant myeloid cells

**Figure 4:**
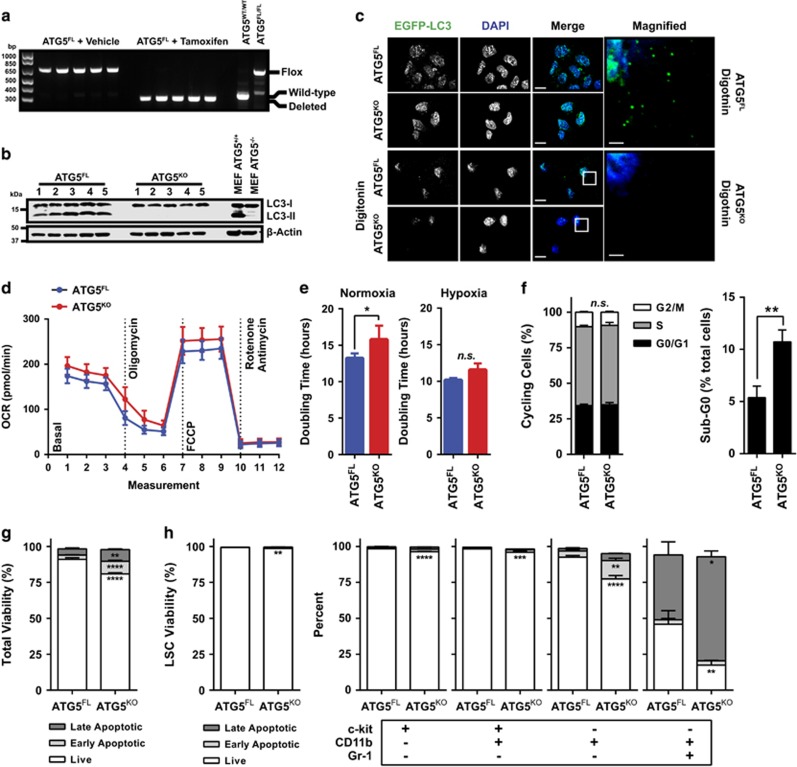
Autophagy, proliferation, and apoptosis of *in vitro* cultured Atg5^FL^ and Atg5^KO^ cells. (**a**) Genomic DNA extracted from the splenocytes of five moribund mice in each group from Figure 3b were analyzed by PCR for the status of Atg5 alleles. DNA extracted from ears of mice with the genotype of Atg5^WT/WT^ and Atg5^FL/FL^ was used as controls. (**b**) Atg5^FL^ and Atg5^KO^ primary splenocytes from panel (**a**) were cultured *in vitro* and treated with 100 nM BafA1 for 2 h, followed by immunoblot analysis. (**c**) Atg5^FL^ and Atg5^KO^ AML cells from panel (**b**) were respectively pooled. Slides were either immediately fixed or subjected to digitonin treatment followed by fixation. Slides were then analyzed by immunofluorescence microscopy of GFP-LC3 (green) and DAPI (blue) on the Olympus IX81 deconvolution microscope with × 100 oil immersion objective lens and processed on the Intelligent Imaging Solutions SlideBook 5.0 software. Ten fields of view were analyzed for each group and representative images are shown. Scale bars in merge images represent 10 *μ*m. White boxes in merged images are shown magnified to the right. Scale bars in magnified images represent 2 *μ*m. (**d**) Atg5^FL^ and Atg5^KO^ cells were analyzed for mitochondrial respiration and presented as oxygen consumption rate (OCR). Error bars represent the S.E.M. of five different clones. (**e**) Five clones each of Atg5^FL^ and Atg5^KO^ cells were seeded at 1 × 10^4^ cells/well in 96-well plates under normoxia and hypoxia, and cell viability was measured every 12 h. Doubling time was calculated according to the exponential growth equation. Statistics were calculated by Student's *t*-test. *P<0.05 (**f**) AML cells were subjected to flow cytometric cell cycle analysis and shown as a percentage of all cells excluding Sub-G0 cells on the left. Sub-G0 cells according to cell cycle analysis are shown as a percentage of all cells. (**g** and **h**) The viability of AML cells in panel (**g**), c-kit^+^Sca-1^−^CD16/32^+^CD34^−^ LSCs, and AML cells of the indicated phenotypes in panel (**h**) were analyzed by flow cytometry and categorized as viable (Annexin V^−^7-AAD^−^), early apoptotic (Annexin V^+^7-AAD^−^), and late apoptotic (Annexin V^+^7-AAD^+^) as a percentage of all cells of the indicated phenotype. Error bars represent the S.D. of five clones in each group. Statistical calculations were performed using ANOVA with multiple comparisons for panels (**e**–**h**). *P<0.05; **P<0.01; ***P<0.001; ****P<0.0001

We sought to confirm that tamoxifen treatment to Atg5^FL^ cells resulted in functional ablation of autophagy in three ways, by PCR, immunoblotting, and immunofluorescence. The status of the Atg5 alleles was examined by PCR in primary splenocytes from moribund Atg5^FL^ mice treated with vehicle (Atg5^FL^) or tamoxifen (Atg5^KO^) (Figure 3b) and confirmed that tamoxifen treatment efficiently deleted Atg5 (Figure 4a). In order to determine whether Atg5 deletion resulted in functional inhibition of LC3 lipidation *in vitro*, we treated both Atg5^FL^ and Atg5^KO^ cells with BafA1 to detect LC3-II by immunoblotting. Under BafA1 treatment, an accumulation of LC3-II was observed in Atg5^FL^ cells but was absent in Atg5^KO^ MLL-AF9 leukemic cells, suggesting that Atg5 is functionally ablated (Figure 4b). We then examined puncta formation by the GFP-LC3 reporter in Atg5^FL^ and Atg5^KO^ MLL-AF9 leukemia cells by fluorescence microscopy. Digitonin treatment prior to fixation facilitates the visualization of GFP-LC3 puncta by releasing cytoplasmic soluble GFP-LC3.^7, 26^ Under BafA1 treatment, a significant accumulation of GFP^+^ puncta was observed in Atg5^FL^ cells with digitonin treatment (Figure 4c). In contrast, digitonin-treated Atg5^KO^ cells demonstrated a lack of GFP signal. These three lines of evidence collectively indicate that LC3 lipidation was functionally ablated in Atg5^KO^ cells.

As autophagy is important for the clearance of damaged mitochondria,^8^ we tested whether Atg5^KO^ cells have altered mitochondrial respiration. Surprisingly, we did not detect a difference in basal respiration or spare respiratory capacity between Atg5^FL^ and Atg5^KO^ cells (Figure 4d). We detected no difference in the extracellular acidification rate (ECAR) during this assay, suggesting that lactate production might not be changed in Atg5-deficient cells (data not shown). The BM microenvironment is hypoxic,^27^ and hypoxia is a known inducer of cytoprotective autophagy.^28^ We therefore assayed whether autophagy contributes to the *in vitro* proliferation of Atg5^FL^ and Atg5^KO^ AML cells under both normoxic and hypoxic conditions. Interestingly, both Atg5^FL^ and Atg5^KO^ cells proliferated more rapidly under hypoxia through unknown mechanisms (Figure 4e). We observed a marginal but statistically significant delay in the proliferation of Atg5^KO^ cells compared with Atg5^FL^ cells under normoxia with a doubling time of 13.3 and 16.2 h, respectively. However, no significant difference in proliferation was observed under hypoxia with a doubling time of 10.2 and 11.6 h, respectively. Correspondingly, no differences were observed in the cell cycle of *in vitro* cultured Atg5^FL^ and Atg5^KO^ cells (Figure 4f). We instead noted an increase in the percentage of Sub-G0 cells in Atg5^KO^ cells, indicating that the apoptosis program could be altered in leukemia cells lacking autophagy. Indeed, flow cytometric analysis of cells stained with Annexin V and 7-AAD confirmed that leukemic cells lacking autophagy demonstrated an increased proportion of cells with ongoing apoptosis (Figure 4g).

AML is a hierarchical malignancy originating from LSCs, and a compromise in the viability of LSCs could explain the mechanism by which Atg5 deletion prolongs the survival of mice burdened with MLL-AF9-driven AML during primary transplantation. Surprisingly, only a very marginal decrease in the viability of LSCs was detected for *in vitro* cultured Atg5^KO^ cells compared with control (Figure 4h). Apoptosis in both c-kit^+^ and c-kit^−^ myeloid populations was compared between Atg5^FL^ and Atg5^KO^ AML cells and was observed to be enhanced in c-kit^−^ myeloid cells, which are differentiated cells that lack leukemia-initiating potential (Figure 4h).^2^ This data collectively suggest that autophagy-deficient AML cells demonstrate enhanced apoptosis in differentiated malignant leukemia cells originating from LSCs *in vitro*.

### Atg5 deletion after secondary transplantation does not alter the chemotherapy response of MLL-AF9-driven AML

**Figure 5:**
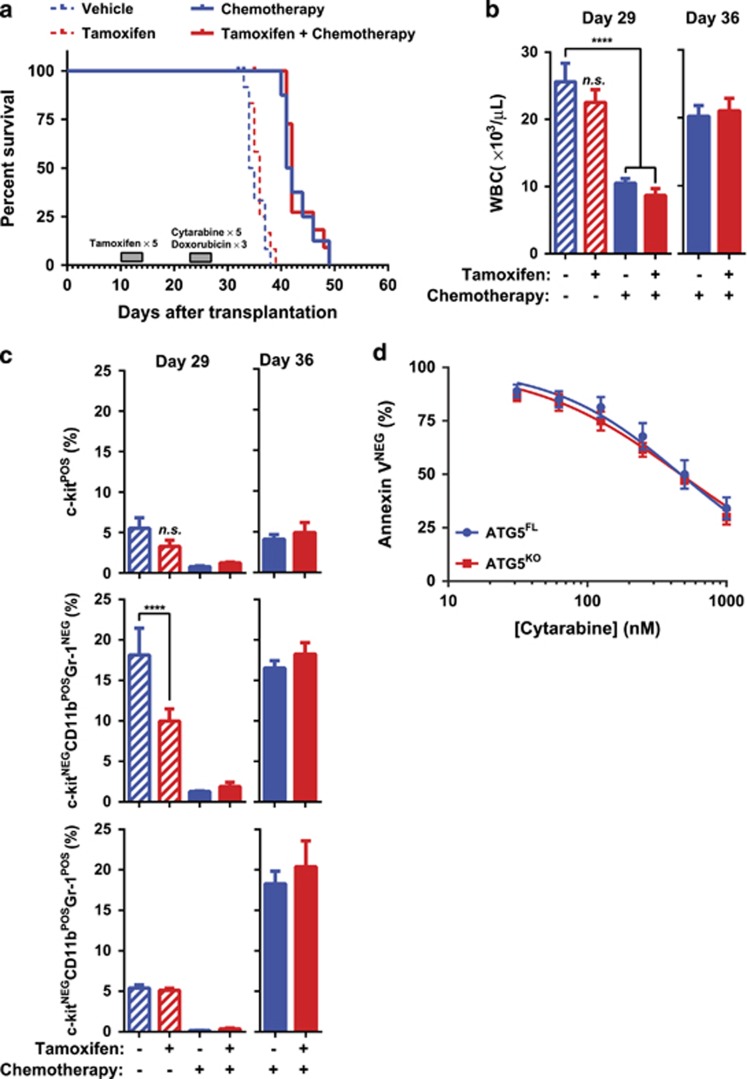
The role of Atg5 in progression and chemotherapy response of MLL-AF9-driven murine AML. (**a**) The Kaplan–Meier survival curve of non-irradiated C57BL/6J recipient mice transplanted with malignant Atg5^FL^ AML cells. Gray boxes indicate the dates by which the indicated treatments or vehicle controls were given once daily. (**b**) WBC counts as enumerated by peripheral blood flow cytometry of mice from panel (**a**) at the indicated dates. Error bars represent the S.E.M. of 10 mice. Statistics were calculated by ANOVA with multiple comparisons. ****P<0.0001; NS, not statistically significant. (**c**) Peripheral blood flow cytometric analysis of mice from panel (**a**) on the indicated days shows the Annexin V^−^7-AAD^−^ cells of the indicated phenotype as a percentage of total Annexin V^−^7-AAD^−^CD45^+^ cells. Error bars represent S.E.M. of 10 mice. Statistics were calculated by ANOVA with multiple comparisons. ****P<0.0001; NS, not statistically significant. (**d**) Atg5^FL^ or Atg5^KO^ cells were treated with the indicated concentrations of cytarabine for 24 h and subjected to flow cytometric analysis, representing Annexin V^−^ cells as a percentage of all cells and normalized to untreated controls. Error bars indicate S.D. of five different clones

The potential of functional autophagy inhibition as a therapeutic strategy has not yet been determined in MLL-AF9-driven AML. We chose to mimic autophagy inhibitor treatment by treating animals with tamoxifen *in vivo* after transplantation of malignant Atg5^FL^ cells that were not previously exposed to tamoxifen. In this secondary transplant model, no significant difference in survival was observed between vehicle- and tamoxifen-treated mice, with median survivals of 34.5 and 36 days, respectively (Figure 5a). However, a significant decrease in the frequency of c-kit^−^CD11b^+^ myeloid cells were observed in the peripheral blood of tamoxifen-treated mice during progression (Figure 5c), consistent with changes observed during *in vitro* culture (Figure 4g).

Autophagy has been postulated as a mechanism of chemotherapy resistance in AML.^29^ Therefore, autophagy's role in chemotherapy response was also determined *in vivo* during secondary transplantation. Following vehicle or tamoxifen treatment, animals bearing malignant AML were administered with either PBS or a chemotherapy regimen with cytarabine and doxorubicin, mimicking the treatment of patients at diagnosis presenting malignant AML.^5^ We observed that MLL-AF9-driven AML responded to chemotherapy, as both vehicle- and tamoxifen-treated mice receiving chemotherapy demonstrated prolonged survival compared with controls (Figure 5a). Additionally, chemotherapy treatment drastically reduced the WBC counts of animals 1 day following treatment termination (Figure 5b). However, no differences were observed between vehicle- and tamoxifen-treated animals in survival (41.5 and 42 days, respectively), WBCs, or myeloid markers during progression or at end point (Figures 5a–c, data not shown). We confirmed that Atg5^FL^ and Atg5^KO^ malignant AML cells responded similarly to chemotherapy *in vitro* (Figure 5d, Table 1). These data indicate that autophagy may not contribute to the chemotherapy response of MLL-AF9-driven murine AML.

Several targeted therapies are being investigated for the treatment of AML and we evaluated whether the potency of these agents could be enhanced by autophagy deletion. As autophagy is important for the clearance of mitochondria,^8^ we tested whether cells lacking autophagy were sensitized to agents which activate mitochondrial apoptosis. Both Atg5^FL^ and Atg5^KO^ MLL-AF9-driven AML cells cultured *in vitro* were completely resistant to Bcl-2 and Bcl-xL inhibitors ABT-199 and ABT-737 (Table 1).^30, 31^ On the other hand, Atg5^KO^ leukemic cells were sensitized to maritoclax, a small molecular antagonist of Mcl-1.^32, 33, 34^ We also observed a significant sensitization of Atg5^KO^ leukemic cells to vorinostat, a histone deacetylase inhibitor, similar to previous findings.^35^

## Discussion

Numerous studies have now described autophagy as essential for hematopoietic homeostasis, and several have suggested an important role for autophagy in myeloid differentiation or proliferation.^9, 13, 36^ Studies have revealed that both human and mouse HSCs demonstrate high levels of autophagic flux.^13, 37^ Indeed, the deletion of key autophagy protein FIP200 in mouse hematopoietic cells abrogated the self-renewal of fetal HSCs, leading to severe anemia and perinatal lethality.^10^ Similarly, knocking down ATG5 and ATG7 function in human CD34^+^CD38^−^ HSCs also drastically reduced their frequency,^37^ and deletion of Atg5 or Atg7 in murine HSCs has also been demonstrated to abrogate the capacity of HSCs to self-renew.^9, 13^ The critical role of autophagy in hematopoiesis as well as the dysregulation of autophagy genes in human AML^13, 14^ strongly implicate its role in AML pathogenesis. Consistent with these studies, our data support a role for Atg5-dependent autophagy in the initiation of MLL-AF9-driven murine AML. By deleting Atg5 *in vitro*, we detected a transient decrease in the colony-forming potential of BM cells immediately following introduction of the MLL-AF9 oncogene (Figure 1g) and observed a statistically significant survival advantage *in vivo* for mice harboring leukemia cells with deleted Atg5 relative to controls during primary transplantation (Figures 2 and 3).

Although we clearly demonstrate the contributions of autophagy in leukemia initiation, autophagy was not involved in the viability of LSCs or maintenance of AML during secondary transplantation (Figures 4h and 5a–c). The specific contribution of Atg5-dependent autophagy to AML initiation but not to progression could be due to several possibilities. First, it is possible that autophagy pathways independent of LC3 lipidation could have a compensatory role in this model of AML.^38, 39^ Studies have shown that mice lacking Atg5 or Atg7 in HSCs demonstrate temporary myelomonocytic proliferation with aberrant maturation.^9, 13^ In this model of MLL-AF9-driven AML, LSCs resemble immature myelomonocytes.^2, 4, 40^ A separate study has already suggested that myeloid cells might rely on Atg5-independent autophagy for survival and differentiation.^41^ Second, MLL-AF9 expression might attenuate autophagy's role as a tumor suppressor by inactivating p53. MLL fusion oncogenes have previously been described to functionally suppress p53.^42, 43^ In pancreatic tumors, autophagy's role in suppressing malignant transformation has been linked to p53 function. Abrogating p53 resulted in a metabolic shift within tumors, reprogramming autophagy to a pro-tumorigenic role.^44^

To our knowledge, we are the first to describe a role of Atg5-dependent autophagy in the initiation of MLL-AF9-driven AML. This is in some contrast to a recent study by Watson *et al.*,^13^ who report an important role for Atg5 in murine MLL-ENL-driven AML. The *in vitro* homozygous deletion of Atg5 following introduction of MLL-ENL oncogene led to significant cell death, suggesting that functional autophagy is essential to maintain MLL-ENL-driven LSCs. In contrast, in our model using MLL-AF9-driven leukemia with homozygous Atg5 deletion, we detected a transient decrease in colony-formation potential during leukemia initiation and no significant LSC cell death hereafter (Figures 1g and 4h). Apoptosis was increased in differentiated myeloid cells originating from malignant LSCs lacking Atg5 (Figures 4h and 5c). Nonetheless, this difference in differentiated myeloid cells was not reflected at end point in any of our studies and did not correlate with survival. Thus the role of autophagy in the survival of differentiated myeloid cells originating from LSCs is unlikely related to its roles in the development of MLL-AF9-driven AML. Watson *et al.*^13^ further observed that heterozygous deletion of Atg5 facilitated AML initiation and development. In these cells, increased *in vitro* and *in vivo* proliferation of autophagy knockdown cells was accompanied by increased glycolysis that is likely due to reprogrammed mitochondrial function, evidenced by increased mitochondrial spare capacity. Although autophagy did have a role in the initiation of AML in our model, we did not observe a difference in the mitochondrial spare capacity or ECAR of malignant MLL-AF9 cells with homozygously deleted Atg5 (Figure 4d). Thus it is unlikely that Atg5 deletion reprogrammed mitochondrial respiration in this MLL-AF9-driven model of murine AML as it did in MLL-ENL-driven AML.

Autophagy is known to participate in treatment response of AML but its roles are controversial.^45^ Autophagy is traditionally implicated in therapy resistance in leukemia, as suppressing autophagy might lead to mitochondrial dysfunction and reactive oxygen species (ROS) production to enhance treatment response.^35, 46, 47^ On the other hand, several studies have shown that autophagy induction enhances treatment response.^48, 49^ One proposed mechanism by which autophagy induction could enhance therapeutic response involves the apparent ability of autophagy to degrade key pro-tumorigenic proteins, including Flt3, PML-RARA, and Bcl-Abl.^49, 50, 51, 52^ In our murine model of MLL-AF9-driven AML, Atg5-dependent autophagy was dispensable for *in vitro* and *in vivo* treatment response to the chemotherapeutics cytarabine and doxorubicin (Figure 5). However, AML cells lacking Atg5 were sensitized to maritoclax and vorinostat treatment *in vitro* (Table 1). Autophagy deficiency has previously been described to enhance vorinostat sensitivity through ROS production.^35^ However, cytarabine^53, 54^ and maritoclax^34^ have also been described to facilitate ROS production to induce cell death, making this an unlikely mechanism by which autophagy protects against maritoclax and vorinostat cytotoxicity. AML cells have previously shown to be dependent on Mcl-1 for survival,^55^ and it is possible that Atg5-deficient AML cells demonstrate increased Mcl-1 dependency, an idea which could be further explored.

Taken together, our study provides insight as to the role of Atg5-dependent autophagy in the development of MLL-AF9-driven murine AML. The *in vitro* deletion of Atg5 transiently decreased the colony-forming capacity of BM cells expressing MLL-AF9, consistent with *in vivo* data where Atg5 deletion significantly delayed MLL-AF9-induced AML initiation. Atg5 deficiency in malignant AML cells was also observed to promote apoptosis in differentiated malignant myeloid cells. Conversely, Atg5-mediated autophagy was not involved in the maintenance or chemotherapeutic sensitivity of malignant AML. The role of autophagy in the pathogenesis of AML remains for further investigation in order to determine whether and how autophagy should be modulated in AML for therapeutic benefit.

## Materials and Methods

### Animal studies

All animal studies were approved and followed the Penn State College of Medicine IACUC guidelines. C57BL/6J and B6.129-*Gt(ROSA)26Sor*^*tm1(cre/ERT2)Tyj*^/J^56^ mouse strains were purchased from Jackson Laboratories(Sacramento, CA, USA), and the B6.129S-*Atg5*^*tm1Myok*^ mouse strain^57^ was obtained from RIKEN Bioresource Center, Ibaraki, Japan. All animals were bred at the Penn State College of Medicine and genotyped as previously described.^56, 57^ Age- and sex-matched animals were used for all animal studies, and all studies were carried out without blinding. Sample sizes were chosen by simulation to ensure adequate power to detect a median survival difference of 5 days.

Primary transplantation studies were largely performed as previously described.^2^ Briefly, magnetically sorted (Miltenyi no. 130-091-224; Bergisch Gladbach, Germany) c-kit^+^ BM cells were spinoculated with concentrated Ecotropic retrovirus with 8 *μ*g/ml polybrene at 1400 × *g* for 2 h at 32 °C once per day for 2 days. Cells were then seeded to methylcellulose medium (Stemcell Technologies no. 03534; Vancouver, British Columbia, Canada) at 1 × 10^4^ cells/ml for three passages of 5 days each. The cells were next transplanted intrafemorally to 500 cGy sublethally irradiated recipient mice under ketamine/xylazine anesthesia at 5 × 10^5^ cells per animal. When applicable, animals were randomized to groups based on transplantation time. Animals suffering from labored breathing, lethargy, or any other signs of morbidity were defined as end point and killed by CO_2_ asphyxiation followed by cervical dislocation and necropsy.

For secondary transplantation, primary splenocytes from primary transplanted leukemic mice were incubated with red blood cell (RBC) lysis buffer (15.5 mM NH_4_Cl, 1.2 mM NaHCO_3_, 10 *μ*M Na_2_EDTA, pH 7.2) for 15 min at 4 °C. Splenocytes were then washed twice and cryopreserved in 90% FBS and 10% DMSO in liquid nitrogen. Splenocytes from at least three different mice were thawed and pooled. Live cells were isolated by centrifugation with Histopaque-1119 (Sigma-Aldrich T5648; St. Louis, MO, USA) at 400 × *g* for 30 min at 24 °C. In all, 200 000 cells were then intrafemorally transplanted to non-irradiated recipient mice.

*In vivo* luminescence imaging was performed using the IVIS Lumina Series III (PerkinElmer; Waltham, MA, USA). Animals were intraperitoneally injected with 150 mg/kg of D-luciferin in PBS and imaged under isoflurane anesthesia. Images were normalized with Living Image V4.1 (PerkinElmer), and the total flux (p/s) of the whole animal was used for quantification.

### Drug treatment

Free-base tamoxifen (Sigma-Aldrich T5648) was prepared at 20 mg/ml and administered by oral gavage at 200 mg/kg as previously described.^58^ Cytarabine (LKT Laboratories C9778; St. Paul, MN, USA) was dissolved in PBS at 20 mg/ml, and doxorubicin (Selleck Chemicals S1208; Houston, TX, USA) was dissolved in PBS and 1% DMSO at 600 *μ*g/ml. Cytarabine and doxorubicin were administered to animals intraperitoneally as previously described.^5^ Cells treated with 100 nM BafA1 (LCL Labs B-1080; Woburn, MA, USA) were analyzed by immunoblotting as previously described.^59^ Cells were treated with 100 nM of the Z isomer of 4OHT (LKT Laboratories H9711) to delete Atg5. Cells were treated with ABT-199 (LKT Laboratories A0776), ABT-737 (LKT Laboratories A0778), maritoclax,^33^ and vorinostat (LKT Laboratories V5734) in 0.5% DMSO for 48 h and subjected to flow cytometric analysis.

### Plasmids and cell culture

The pMSCV-luc-IRES-YFP plasmid was received from Dr Gerard Grosveld (St. Jude Children's Research Hospital). The construction of the pMSCV-MLL-AF9-EF1*α*-luc-P2A-GFP-LC3 plasmid was carried out by replacing the luc-IRES-YFP open reading frame in the pMSCV-luc-IRES-YFP vector with the following components: MLL-AF9 from pMIG-MLL-AF9 (Dr Robert Paulson, Penn State University Park, PA, USA); EF1*α* from pCDH1-MCS-EF1-Puro (System Biosciences CD510B; Palo Alto, CA, USA); Luciferase from pMSCV-luc-IRES-YFP; P2A from pULTRA^60^ (Dr Malcolm Moore, Memorial Sloan Kettering, Addgene (Cambridge, MA, USA) no. 24129); GFP-LC3 from pMXs-IP-EGFP-LC3^61^ (Dr Noboru Mizushima, University of Tokyo, Tokyo, Japan).

The Atg5^WT/WT^ and Atg5^−/−^ mouse embryonic fibroblasts were kindly provided by Dr Noboru Mizushima (Tokyo Medical and Dental University, Tokyo, Japan) and cultured in DMEM medium with 20% FBS and 1% antibiotic–antimycotic solution (Mediatech, Manassas, VA, USA). Primary murine leukemia and hematopoietic cells were cultured in IMDM supplemented with 20% FBS, 1% antibiotic-antimycotic solution (Mediatech), 50 ng/ml SCF (PeproTech 250-03; Rocky Hill, NJ, USA), 20 ng/ml IL-3 (PeproTech 213-13), and 10 ng/ml IL-6 (PeproTech 216-16) and maintained between 0.2 and 1.0 × 10^6^ cells/ml unless otherwise stated. For luminescence studies, luminescence was measured immediately after adding 75 *μ*g/ml D-luciferin to cell culture on the BMG ClarioStar (BMG Labtech; Ortenberg, Germany).

### Colony-formation assay

Cells were seeded at 500 viable cells/ml, according to the trypan blue exclusion assay, to methylcellulose medium (Stemcell Technologies no. 03534) and cultured for 6 days before manual counting of colonies under light microscopy. For serial plating, cells were washed twice before re-seeding at 500 viable cells/ml to fresh methylcellulose medium. For 4OHT treatment, 100 nM 4OHT or 0.1% ethanol (vehicle) was added to methylcellulose medium before cells were seeded.

### PCR

Genomic DNA was extracted from primary splenocytes using the DNeasy Blood and Tissue Kit (Qiagen no. 69504; Hilden, Germany) according to the manufacturer's recommendations. PCR was then performed with 10 ng of genomic DNA, Perfect Taq Plus MasterMix (5PRIME no. 2200095; Gaithersburg, MD, USA) and previously published primers^57^ to detect wild-type, floxed, and deleted Atg5 alleles.

### Histology

Tissues were fixed in 10% neutral-buffered formalin for 24 h and then stored in 70% ethanol. Soft tissues were mounted in paraffin, sectioned, and stained by hematoxylin and eosin. Peripheral blood smears were fixed with methanol for 1 min. May Grünwald–Giemsa stain was performed using the May Grünwald solution (Sigma-Aldrich 32856) and the modified Giemsa stain (Sigma-Aldrich GS1L) according to the manufacturer's recommendations.

### Flow cytometry

Peripheral blood was collected by cardiac puncture from moribund mice during necropsy. BM was flushed from the femur and tibia with a 27-gauge needle, and the spleen was dissociated through 40 *μ*m nylon mesh. For live animals, peripheral blood was collected by saphenous vein puncture into the Safe-T-Fill EDTA capillary blood collection system (RAM Scientific no. 077051; Yonkers, NY, USA). RBCs were lysed in RBC lysis buffer for 15 min on ice for spleen and BM or at room temperature for 20 min for peripheral blood. Cells were washed and blocked with either 2% unlabeled mouse CD16/32 (BioLegend no. 101302; San Diego, CA, USA) or 0.5% of BV711-CD16/32 (BioLegend no. 101337) in flow cytometric buffer (PBS, 2% FBS, 100 *μ*M EDTA, 0.1% sodium azide) for 10 min on ice. Cells were then labeled with fluorochrome-conjugated antibodies listed below for 15 min on ice. Cells were washed twice with flow cytometric buffer and fixed with Fixation Buffer (BioLegend no. 420801) on ice for 20 min. Cells were washed twice and analyzed immediately on the BD Fortessa flow cytometer (BD, Franklin Lakes, NJ, USA) or stored in 90% FBS and 10% DMSO at −80 °C for later analysis.

The following antibodies were used at the indicated concentrations in this study: Annexin V/FITC, 1:50 (BD no. 556420); 7-AAD, 1:20 (BD no. 559925); CD11b/BV711, 1:200 (BioLegend no. 101241, San Diego, CA, USA); Ly-71/BV605, 1:200 (BioLegend no. 101241); CD19/BV650, 1:100 (BioLegend no. 115541); Gr-1/AF700, 1:200 (BioLegend no. 108422); TER-119/APC-Cy7, 1:200 (BD no. 560509); CD3/BV785, 1:200 (BioLegend no. 100231); c-kit/PE, 1:200 (BioLegend no. 105807); CD45/BV421, 1:50 (BioLegend 103133); Lineage Cocktail/V450, 1:20 (BD no. 561301); CD48/PE-Cy7, 1:200 (BD no. 560731); Sca-1/APC, 1:200 (BioLegend no. 108111); CD150/BV605, 1:200 (BioLegend); and CD34-AF700, 1:50 (BD no. 560518).

For cell cycle analysis, cells were fixed with 70% ethanol overnight at 4 °C. Cells were then washed twice in flow cytometric buffer and resuspended in flow cytometry buffer with 50 *μ*g/ml propidium iodide (BioLegend no. 421301) and 20 *μ*g/ml RNase A (Qiagen no. 19101) for flow cytometric analysis.

### Immunofluorescence microscopy

Exponentially growing cells were prepared on microscope slides through the Cytospin 4 Cytocentrifuge (Thermo Scientific, Leesport, PA, USA) at 100 000 cells/well. Slides with digitonin treatment were incubated with 250 *μ*g/ml digitonin for 2 min and washed with PBS. Slides were fixed in 4% paraformaldehyde in PBS at room temperature for 20 min and washed three times with PBS. Slides were then mounted in Prolong Gold mounting medium with DAPI (Thermo Scientific no. P36941), and immunofluorescence microscopy was performed as previously described.^59^

### Mitochondrial stress test

A total of 100 000 cells were seeded in XF medium supplemented with 4.5 g/l glucose and 2 mM L-glutamine to the XF96 cell culture plate coated with Cell-Tak (Corning no. 354240, Manassas, VA, USA) according to the manufacturer's recommendations. The assay was run on the XF96e Flux Analyzer (Seahorse, Santa Clara, CA, USA) with 1 *μ*M of the indicated compounds added to the cells from the XF Cell Mito Stress Test Kit (Seahorse) according to the manufacturer's recommendations.

### Cell proliferation assay

In all, 10 000 cells were seeded per well to 96-well plates in 100 *μ*l of IMDM supplemented with 20% FBS, 1% antibiotic–antimycotic solution, 50 ng/ml SCF, 20 ng/ml IL-3, and 10 ng/ml IL-6. Cells under hypoxia treatment were incubated in the humidified InvivO_2_ 300 Hypoxia Workstation (Baker Ruskinn, Pencoed, UK) at 1.8% O_2_, 5% CO_2_, and 37 °C. At the indicated time points, 10 *μ*l of PrestoBlue reagent (Thermo Scientific no. A13262) was added and incubated for 1 h at 37 °C. Fluorescence was measured according to the manufacturer's recommendations on the BMG ClarioStar.

### Statistical analysis

Sample sizes were estimated with power calculations. All statistics were performed as indicated by GraphPad Prism (version 6.00 for Windows, GraphPad Software, La Jolla CA, USA).

## Figures and Tables

**Table 1:** Sensitivity of Atg5^FL^ and Atg5^KO^ AML cells to the indicated small molecules represented as EC_50_ for the induction of Annexin V^+^ apoptosis

	**IC**_**50**_ **(nM)**		
	**ATG5**^**FL**^	**ATG5**^**KO**^	***P*****-value**
Cytarabine	163.8	160.0	0.8185
ABT-199	>1000	>1000	NA
ABT-737	>1000	>1000	NA
Maritoclax	9616	6248	**0.0035
Vorinostat	9031	6162	**0.0013

**P-value <0.005.

## Abbreviations

4OHT: 4-hydroxytamoxifen
AML: acute myeloid leukemia
ANOVA: analysis of variance
BafA1: bafilomycin A1
BM: bone marrow
CreERT2: tamoxifen-inducible Cre recombinase
dMLL-AF9: dual-promoter/reporter MLL-AF9 retroviral vector
ECAR: extracellular acidification rate
EF1*α*: elongation factor-1 alpha
HSC: hematopoietic stem cell
MSCV: murine stem cell virus
RBC: red blood cell
ROS: reactive oxygen species.
